# Online Food Security Discussion Before and During the COVID-19 Pandemic in Native Hawaiian and Pacific Islander Community Groups and Organizations: Content Analysis of Facebook Posts

**DOI:** 10.2196/40436

**Published:** 2022-09-30

**Authors:** Cassandra Jean Nguyen, Christian Pham, Alexandra M Jackson, Nicole Lee Kamakahiolani Ellison, Ka`imi Sinclair

**Affiliations:** 1 Institute for Research and Education to Advance Community Health Washington State University Seattle, WA United States; 2 Elson S Floyd College of Medicine Washington State University Spokane, WA United States

**Keywords:** social media, oceanic ancestry group, food insecurity, social networking, COVID-19, Facebook, community, Hawaiian, Pacific Islander, online, food, risk factor, disease, cardiometabolic, diabetes, hypertension, food security, digital, support, culture

## Abstract

**Background:**

The Native Hawaiian and Pacific Islander (NHPI) population experiences disproportionately higher rates of food insecurity, which is a risk factor for cardiometabolic diseases such as cardiovascular disease, type 2 diabetes, obesity, and hypertension, when compared to white individuals. Novel and effective approaches that address food insecurity are needed for the NHPI population, particularly in areas of the continental United States, which is a popular migration area for many NHPI families. Social media may serve as an opportune setting to reduce food insecurity and thus the risk factors for cardiometabolic diseases among NHPI people; however, it is unclear if and how food insecurity is discussed in online communities targeting NHPI individuals.

**Objective:**

The objective of this study was to characterize the quantity, nature, and audience engagement of messages related to food insecurity posted online in community groups and organizations that target NHPI audiences.

**Methods:**

Publicly accessible Facebook pages and groups focused on serving NHPI community members living in the states of Washington or Oregon served as the data source. Facebook posts between March and June 2019 (before the COVID-19 pandemic) and from March to June 2020 (during the COVID-19 pandemic) that were related to food security were identified using a set of 36 related keywords. Data on the post and any user engagement (ie, comments, shares, or digital reactions) were extracted for all relevant posts. A content analytical approach was used to identify and quantify the nature of the identified posts and any related comments. The codes resulting from the content analysis were described and compared by year, page type, and engagement.

**Results:**

Of the 1314 nonduplicated posts in the 7 relevant Facebook groups and pages, 88 were related to food security (8 in 2019 and 80 in 2020). The nature of posts was broadly classified into literature-based codes, food assistance (the most common), perspectives of food insecurity, community gratitude and support, and macrolevel contexts. Among the 88 posts, 74% (n=65) had some form of engagement, and posts reflecting community gratitude and support or culture had more engagement than others (mean 19.9, 95% CI 11.2-28.5 vs mean 6.1, 95% CI 1.7-10.4; and mean 26.8, 95% CI 12.7-40.9 vs mean 5.3, 95% CI 3.0-7.7, respectively).

**Conclusions:**

Food security–related posts in publicly accessible Facebook groups targeting NHPI individuals living in Washington and Oregon largely focused on food assistance, although cultural values of gratitude, maintaining NHPI culture, and supporting children were also reflected. Future work should capitalize on social media as a potential avenue to reach a unique cultural group in the United States experiencing inequitably high rates of food insecurity and risk of cardiometabolic diseases.

## Introduction

### Background

Food security is defined as “enough food for an active, healthy life” for all members of a household [1]. In contrast, a household is considered to be food insecure when the provisions outlined for food security are limited by money or lack of resources [1]. Food insecurity is associated with poorer outcomes in the prevention and management of chronic cardiometabolic diseases such as diabetes, hypertension, obesity, and cardiovascular disease [2-5]. These disease outcomes are a product of the compensatory behaviors that members of food-insecure households employ to avoid hunger, which can include relying on nutritionally poor, calorie-dense foods [6].

The prevalence of food insecurity in the United States has increased during the COVID-19 pandemic, impacting an estimated 54 million households [7], as many household members experienced periods of under- or unemployment. In attempts to ensure that US households had access to food, many nutrition assistance programs evolved during the COVID-19 pandemic. These evolutions included the rapid expansion of the US Department of Agriculture’s pilot program for Supplemental Nutrition Assistance Program (SNAP) benefits to be used online [8]. As internet and social media play a growing role in how individuals access food and nutrition information [9], it is important to consider how the food environment—defined as the interface where people interact with the wider food system—becomes increasingly digital [10]. A scoping review of studies of the digital food environment demonstrated how social networking platforms can be used to shape food culture, drive trends, and also serve as an abundant source of health and nutrition information [9].

Social networking platforms, and the online communities they foster, have been capitalized on in various ways in efforts to address chronic cardiometabolic diseases and food insecurity. Facebook is one of the largest online social networking platforms, which was used by over 2 billion people each month in 2019 and 2020 [11]. Facebook has been used to facilitate emotional and informational support via peer-to-peer and caregiver-to-patient interactions, leading to improved disease self-management among patients with diabetes [12]. However, these approaches to focus on cardiometabolic diseases using social media do not appear to have yet been adapted to address food insecurity. In a scoping review of 39 studies of digital technology use in food assistance programs, only a single study described the use of social media by a food pantry to communicate with clientele [13]. Another single observational study found that consuming information about COVID-19 online was associated with individuals’ concerns about food insecurity [14]; however, it remains uncertain how social media may be used to discuss, and ultimately address, food insecurity.

Native Hawaiian and Pacific Islander (NHPI) communities are a priority population for consideration as the digital food environment continues to evolve in the United States. NHPI adults experience a disproportionately higher prevalence of cardiovascular disease, diabetes, hypertension, and obesity compared to other demographic groups [15,16]. Additionally, a recent study showed that NHPI adults experience a higher prevalence of food insecurity (21%) than their non-Hispanic white counterparts (8%) [17]. A population-based survey of primarily NHPI adults (N=637) in low-income households found that very low food security was strongly associated with greater odds of both hypertension and diabetes [18]. Interventions to address these dual burdens of cardiometabolic disease and food insecurity among NHPI adults are needed. NHPI populations have distinct cultural characteristics, including values, history of foreign colonization and exploitation, and emigration to the continental United States, warranting culturally informed interventions [19-21].

### Context of the Study

Healthy Hearts Among Pacific Islanders (HHAPI) is an educational program created by and for NHPI people. The goal of HHAPI is to utilize culturally grounded evidence-based interventions to mitigate cardiometabolic conditions in the NHPI population via self-management of hypertension and diabetes at the individual, family, and policy levels. At the individual and family levels, HHAPI began in 2016 with in-person hypertension management classes hosted in community organizations in the Pacific Northwest, with one of the largest NHPI populations outside of Hawaii [22], which then shifted online in 2020 due to the COVID-19 pandemic. At the policy level, HHAPI initially intended to influence grocery store labeling of foods as either being low in sodium or high in potassium. Given the unanticipated changes with the COVID-19 pandemic, including the growing importance of social media, the HHAPI project shifted to observing the online food environment and discussions of food insecurity among NHPI people living in the Pacific Northwest, as an opportunity to better understand social media as a potential mechanism to decrease food insecurity and cardiometabolic conditions among NHPI adults.

The purpose of this study was to explore the online presence (static website, social media accounts) of NHPI-serving community groups and cultural or health-focused organizations in Washington state, and to compare the nature (eg, educational, event promotion, resource sharing) and frequency of food insecurity–related messages posted on their social media sites from March through June 2019 and from March through June 2020. We also evaluated the amount and type of engagement (ie, likes, shares, comments) on food insecurity–related messages by the nature of the post.

## Methods

### Sample

The sample of online data was extracted from publicly accessible Facebook pages and groups. On Facebook, individuals make posts, comprised of text, image(s), video(s), and/or hyperlink(s) to other webpages, and other individuals can engage with the post. Engagement includes commenting with their own text, image(s), video(s), and/or hyperlink(s), or by clicking the “reaction” button to show one of seven possible reactions (Like, Love, Care, Haha, Wow, Sad, and Angry) as a small, animated symbol listed under the original post [23,24]. Facebook posts can be made on an individual’s profile; a page representing a business, organization, or event; or within a group. Pages for businesses, organizations, or events allow for staff or volunteers to make posts to communicate with their target audience. In contrast, Facebook groups are online spaces that allow for any group members to make posts to communicate around shared interests or identities, allowing for a multidirectional conversation. Facebook groups can be public and visible to any Facebook user, or they can be private, restricted to Facebook users who have applied to join the group and have been formally accepted by group administrators.

Facebook business pages and groups focused on serving NHPI community members in the Pacific Northwest region were identified through consultation with three staff members who identified as Native Hawaiian or Pacific Islander and lived in the Pacific Northwest, a region with large communities of NHPI people. Each group’s Facebook page or group was reviewed to ascertain corresponding details about each organization’s mission or objective. To help maintain confidentiality, the names of the groups and organizations are not included in this manuscript.

### Ethics Approval

The study was certified as “Exempt” from approval by the Washington State University Institutional Review Board (IRB) on March 22, 2021 (IRB #18784).

### Keywords

The list of keywords used to identify posts related to food security was generated in three steps. First, the keywords were identified through a review of related literature [25-29], which included the terms food desert, food insecurity, food security, food supply, food access, food sufficiency, and food insufficiency. Next, the list was supplemented with plain-language synonyms or related terms to include free food, free lunch, free breakfast, free dinner, free plate, free meal, food pick-up, boxed meals, boxed food, food aid, food kitchen, food distribution, meal center, food donation, hunger, and hungry. Lastly, the list of terms was reviewed by a member of the research team with expertise in food security and experience in conducting systematic literature reviews. Based on their suggestion, the final list of keywords included additional terms referring to food assistance programs (food stamp, food bank, food shelf, food pantry, food drive, soup kitchen, food closet, WIC [Special Supplemental Nutrition Program for Women, Infants, and Children], SNAP, EBT [Electronic Benefits Transfer], SNAP-Ed [SNAP Education], senior meals, and school lunch).

### Data Extraction

One research team member searched all identified Facebook pages and groups using each keyword as the search term. The results were restricted to posts made in the months of March through June in the years 2019 and 2020 to compare social media engagement before the COVID-19 pandemic to the same period during the pandemic. The researcher conducting the searches was not a member of any of the Facebook groups or a follower of the Facebook pages searched. All searches were logged and the date of any resulting posts was documented. Each post was then assessed for its relevance to food security. Posts were considered related to food security if they contained information about food assistance opportunities/events or about individual or community needs for food due to income or access constraints. If the post was considered relevant to food security, additional data were documented about the content of the post (ie, text, web links, images, videos) as well as the amount and type of engagement (ie, comments, shares, and reactions). All posts that were not considered relevant were excluded from coding and analysis. These searches were conducted between June and August 2021.

### Qualitative Coding Procedures

To identify and quantify the nature of posts identified, a content analytical approach was used to assess patterns of posts and related engagement by their categorical nature [30]. This process began after all searches were completed between September and October 2021. Three members of the research team independently reviewed all of the relevant posts identified, and created memos that reflected possible codes, corresponding definitions, and example posts from the data set. Two members of the research team reviewed the relevant literature to identify possible literature-based codes that have been used in prior research on food security, public health, or online information exchange. These memos and potential literature-based codes were discussed by the three researchers as a group and used to create an initial codebook that was used by all three researchers to independently code the data. The agreement of these independently coded data was evaluated according to the percentage agreement between coders. Differences in interpretation or application of the codebook during the coding process were evaluated and discussed as a group to refine the codebook and maximize clarity and specificity. The final codebook was used by the group to review and finalize codes for all posts and accompanying comments that characterized the nature of information shared and audience engagement.

### Analysis

To describe the online presence of the NHPI-serving groups and organizations identified, the purpose or mission of the organization’s page or group was broadly categorized. Means and frequencies were used to describe the number of users engaged by each page or group. To assess the nature and engagement of posts, the number of posts identified per page by year and types of engagement (eg, reactions, shares) were enumerated. Codes resulting from the content analysis were described and compared by year, page type, and engagement. Qualitative coding was organized in a spreadsheet (Excel, Microsoft Office 365) and quantitative descriptive analyses were conducted in STATA/MP 17.0 (StataCorp, LP).

## Results

### Data Summary

Seven relevant Facebook pages (n=5, 71%) or groups (n=2, 29%) were identified. Broadly, the stated missions or objectives of these organizations and groups created a welcoming online space to build community among Pacific Islanders, share resources, promote events or local businesses, preserve culture, and/or address social or health inequities. All groups or pages had, on average, 4229 (SD 2731) followers or members. Facebook groups had, on average, a greater number of members (mean 6010, SD 2279) than pages had followers (mean 3516, SD 2769).

Across all groups or pages, 1594 posts were identified using all search terms during the selected time periods. Of these, 278 posts were duplicates (ie, different search terms resulted in the same post being identified) and were removed. Of the 1316 nonduplicated posts, 88 (6.69%) were considered relevant to food security. Nonrelevant posts included announcements for surveys and information about hours of local businesses, among other topics. Among the 88 relevant posts, a subset of posts (n=8) were reposted 2-4 times on different dates and/or in different groups with identical text content. These posts were treated as unique and remained in the data set to reflect their greater potential reach. In 2019, there were 8 relevant posts identified in contrast to 80 relevant posts identified in 2020. Across the 7 groups or pages, a range of 0-45 relevant posts were identified with a mean of 12.6 posts (16.2 SD) per group or page. Of the 88 posts, 12 (14%) had comments associated with the posts, which were qualitatively analyzed and are described below.

### Codebook

A total of 20 codes for posts and 7 codes for comments were developed and used to characterize the data set. Codes were not mutually exclusive, and each post had a mean number of 3.8 (SD 1.7) codes applied. For the posts in 2019, a total of 15 codes were applied (mean of 1.9 per post), whereas the posts in 2020 had a total of 321 codes applied to them (mean of 4.0 per post). Among posts with comments, a mean of 1.8 (SD 0.8) codes were applied to the comments. After coding was complete, the concepts for posts were broadly organized into 6 categories, with some codes represented in more than one category, to assist with interpretation and presentation of results. Given the small number of comments included in the data set, all 7 codes for comments were interpreted and presented together.

### Nature of Posts

#### Literature-Based Codes

The prevalence of codes based on concepts related to food insecurity or studies of social media–based communication were relatively low (Table 1). Nonfood-related needs of households experiencing food insecurity were referenced in 14 posts (16%), educational resources or information were shared in 4 posts (5%), and promotion of businesses was included in 9 posts (10%). It is possible (and likely) that many posts promoting businesses were not captured in this study given the keywords used. Posts referring to nonfood needs of households experiencing food insecurity made mention of social isolation, employment, and housing issues, among others, reflecting the fact that food insecurity does not occur in isolation without other social risks. All education-coded posts were made in 2020, which shared information about policies and programs, including the 2020 Census, the Fresh Bucks program in King County, and the Pandemic EBT program. Business-promoting posts included advertisements for restaurants and farmer’s markets, often referring explicitly to the food(s) being sold and/or incorporating cultural references into the text (Table 1).

**Table 1:** Literature-based codes, definitions, and example posts.

Codes and subcodes	Definition (post text, hashtags, or images)	Example post
Nonfood social risk factors	Refers to other resources or circumstances that are related to food insecurity but not directly about food availability, such as housing, transportation, health, safety, education, income, isolation, and/or employment	…*We will continue to show up for one another especially for those experiencing elevated social isolation, food - housing - employment insecurity, lack of accessibility, support systems and community connection…*
Education	Provides education (or refers to educational resources) to learn more about the issue of food security, and/or opportunities for individuals or households to acquire education about food preparation and/or nutrition	*Due to COVID-19 school closures, families may be eligible to get help with food benefits. These food benefits are called Pandemic EBT^a^* *Emergency School Meals Program or P-EBT^b^. Find out more below. No citizenship requirements and is not considered under public charge.*
Paid event or business promotion	Provides information about businesses or events that require payment to support	*[Zanny face emoticon]* *PLATE SALE WEEK 5️* *[Zanny face emoticon]* *PLATE SALE WEEK 5️$ DAY* *[Victory hand emoticon] [Zanny face emoticon]*
Free meals for kids	Indicating that children are provided meals for free at events (or businesses) that otherwise require payment for food	*School’s out! At [Organization name], we are offering FREE kid meals, all day, every day!*
Food item(s)	Identify specific types or examples of foods (not meal occasions) that are being sold or distributed^c^	*Most King County farmers markets are now open! Have SNAP^d^/EBT? You can use it to buy fruits and veggies at farmers markets! Look for Fresh Bucks signs to get a $1 off for every $1 you spend. Fresh Bucks King County #FreshBucks* *More info on how to use Fresh Bucks: [weblink]*
Cultural context	Directed toward AAPI^e^ subpopulations through direct mention of population(s), mention of cultural consideration and/or traditional/local foods, inclusion of NHPI^f^ language or words, photographs of community members, and/or an AAPI language is mentioned as part of the service/event being described	*When #COVID_19 closed schools, many AAPI students who relied on school lunch & breakfast programs were facing hunger. The Census directs $8.7 BILLION for school meals every year. Get counted to help fight hunger for the next 10 years #AAPI2020 #RootedInCommunity #RisingTogether #APAHM [weblink]*

^a^EBT: Electronic Benefits Transfer.

^b^P-EBT: Pandemic Electronic Benefits Transfer.

^c^Excludes images.

^d^SNAP: Supplemental Nutrition Assistance Program.

^e^AAPI: Asian American and Pacific Islander.

^f^NHPI: Native Hawaiian and Pacific Islander.

#### Food Assistance 

By far, the most common topic (69/88, 78%) of posts was sharing information about food assistance for community members (Table 2). A subset of these posts described foods that would be available, either in broad terms (ie, produce, meat) or listed specifically (ie, katsu chicken, spam, rice). References to NHPI culture, such as through words from NHPI language(s) or pictures of community members, were included in 19% of the 69 posts. Over half of posts about food assistance focused on meeting children’s food needs such as through free lunch or breakfast services. Although these services for children were widely discussed in 2020, possibly due to the rapid expansion of services during school closures, they comprised a greater proportion of posts identified in 2019. Food assistance posts were frequently used in both 2019 and 2020 to share logistical information about events or programs, such as dates, addresses, and eligibility criteria. A unique phenomenon to 2020 was the use of posts (n=11) to share time-sensitive information about unanticipated changes to the timing or availability of food assistance. An additional code that was only present in 2020 was the promotion of free meals for children at restaurants or other food retailers (n=4). Although this served as a subcode to business promotion, most of these posts (n=3, 75%) indicated that no purchases were necessary to receive food for children, suggesting that these offers may be an acceptable way to receive food assistance.

**Table 2:** Food security specific codes, definitions, and example posts.

Codes and subcodes			Definition (post text, hashtags, or images)		Example post
**Food assistance**					
	Food assistance resources	Describe specific events, programs, or other information about food assistance (groceries, meals, and/or lunches) available to individuals and/or children		*!! Stop by today for food boxes. Our food boxes, as usual will be available until supplies last on a first come, first served basis.* *Pick up at our office location in Kent. [Address]*	
	Child food assistance	Food assistance (lunches, meals, groceries, etc) mentioned is specific to (or primarily targeted toward) providing food for children and/or families with children		*Stop by and pick up a FREE lunch and PBS KIDS educational activity packet during school closure.*	
	Confidentiality of assistance	Refer to confidentiality or privacy of the food assistance being offered		*If anyone is having a difficult time getting food to feed yourself and your family, please call the Sunshine Pantry in Beaverton, no questions, phone [phone number].*	
	Logistics of assistance	Provides logistical information about the assistance, such as times/days, location(s), and/or eligibility (or a link or phone number to access this information)^a^		*!! !!Thank you to our volunteers and to everyone that stopped by today for our food distribution. We were able to give away 600 boxes of meat, dairy, and produce. We will continue our food distribution next week Monday up until supplies last. First come first served. So please keep a look out on all our social media platforms for more updates.* *Fa’afetai Tele Lava !!*	
	Updates to food assistance	Provides time-sensitive information about unanticipated changes to the timing and availability (ie, food is gone or event is over) of food assistance within a time frame of 1 week or less^a^		*THAT’s a WRAP!* *Another great big thank you to our community members and committed volunteers for another successful curbside food distribution!* *If you missed us this week, don’t worry, stop by the office next week Monday, 2PM* *[Address]*	
	Food item(s)	Identify specific types or examples of foods (not meal occasions) that are being sold or distributed^b^		*[Location]: Million Pounds of Potatoes giveaway tomorrow (May 14th)* *With the closure of restaurants there is an abundance of potatoes and the [Organization Name] is shifting to help those in need by hosting “On the Road to a Million Pounds of Potatoes” with plans to host their largest potato giveaway to date this Thursday, May 14 at 11am…*	
	Cultural context	Posts are directed toward AAPI^c^ subpopulations through direct mention of population(s), mention of cultural consideration and/or traditional/local foods, inclusion of NHPI^d^ language or words, photographs of community members, and/or an AAPI language is mentioned as part of the service/event being described		…*It is 2nd nature for our communities to move into roles of caring for one another with love, compassion, kindness, and tenderness. We will continue to show up for one another…*	
**Perspectives of food insecurity**					
	Self-disclosure	Refers to personal experiences of food insecurity or related food hardships experienced by user or user’s family members		Not applicable; no relevant content identified	
	Critical jests about food assistance	Presents information or cues specific to food assistance framed as a critique or joke		*Who selling ebt* ^e^ *??? [4 laughing with tears emoticons]* *let me knoo!!! Hahaha [Name] smdh*	
	Feedback	Requests that readers provide feedback or provide stories about their experiences with food security or food assistance on surveys or through other structured data collection^a^		*We want to hear from you!* *Share your story about [Organization Name], and we may use it in a future post. #momstrong #[Organization Name]*	

^a^Excludes hashtags.

^b^Excludes images.

^c^AAPI: Asian American and Pacific Islander.

^d^NHPI: Native Hawaiian and Pacific Islander.

^e^EBT: Electronic Benefits Transfer.

#### Perspectives of Food Insecurity

A minority of identified posts demonstrated how Facebook groups could be used by members to communicate their opinions, perceptions, or personal experiences with food insecurity (Table 2). One post was used to make a joke about others in the community using the SNAP benefits with text, emojis, and an accompanying image. This acknowledged the presence of food insecurity and resulting reliance on governmental food assistance, while making the community members’ relying on these programs the target of the joke. However, this discourse may not be considered hostile, as one group member posted in 2020 that they participated in the free school meal program; during the COVID-19 pandemic, many schools distributed free meals in Washington state to any children, regardless of income. Thus, use of these programs may not have been as stigmatizing as in prior years. No other individuals created posts disclosing their personal experiences of food insecurity. However, one food assistance organization, a clinic affiliated with the WIC joined the discourse online by creating a post that asked individuals to provide stories about their experiences of food insecurity in an anonymous survey.

#### Community Gratitude and Support

Positivity and promotion of community were salient sentiments in identified posts (Table 3). This was noted through explicit expressions of gratitude and thankfulness (n=21), which were directed toward individuals, donors, programs, or the community in general, as examples. Posts were also used to advertise opportunities for individuals to volunteer their time or provide donations (n=12) to support programs such as food drives in the community. One post was created by an individual to redistribute food their household had received from a school-based food assistance program.

**Table 3:** Community- and context-related codes, definitions, and example posts.

Codes and subcodes		Definition (post text, hashtags, or images)	Example post
**Community gratitude and support**			
	Gratitude	Mentions appreciation for support of individuals, organization, or the broader community for support of programs, events, and/organizations	*We are grateful for the opportunity to partner with [Organization Name] and [Organization Name] to provide food meals for our communities in need during the COVID-19 pandemic.*
	Support opportunities	Refer to opportunities for community members to provide support to food security through volunteering, food donations, fundraising, or by attending events that support food banks or other charitable organizations	*[Organization Name] - Take what you need, leave what you can.* *If school closures have you or someone you know worried about access to food, please stop by our front-yard pantry and take what you need.* *If you’d like to help out, please feel free to replenish the pantry as needed. Remember, school is closed until the end of April, so please check in over the next several weeks. We ask that you ONLY restock the shelves that have space. Please do not leave any food outside of the pantry. [Address]*
	Reallocation of resources received	Indicates the user has excess food or related resources received for food security that they would like to give away^a^	*Anyone in da group in da [city] or [city] area wanna take these off our hands? My neighbors and I have so much from da free lunch program at da school n we not gonna make use of it. We drink almond milk n don’t want these to go to waste. Lmk!*
**Macrolevel contexts**			
	COVID-19	Refers to the COVID-19 pandemic explicitly or implicitly and/or ramifications of the pandemic, such as staying healthy, wearing masks, or disruptions to the food system	*We understand that during this time food accessibility can be limited due to vacant grocery stores. Families will be able to pick up a box of Produce, Dairy, or combo of Meat & Produce.*
	Federal policies	Refers to federal policy news updates or short-term political activities that may be related to current or future food security	*The Trump administration’s proposed rule change to food stamp work requirements could leave hundreds of thousands of the most financially vulnerable Americans without the monthly assistance*
Emoticon use to supplement text		Supplemented with the addition of emoticon(s)^a,b^	*Focus on Pacific Islanders but Open to ALL!!!! Get there EARLY while supplies last [4 man running emoticons, wink face emoticon, thumbs up emoticon]* *ALL will receive boxes filled with:* *Produce [emoticons of broccoli, carrot, apple, orange, lemon, pear, banana, avocado] Dairy [emoticons of egg, cheese, glass of milk] Meat [emoticons of meat with bone, poultry leg, steak, and bacon]*

^a^Excludes hashtags.

^b^Excludes images.

#### Macrolevel Contexts

A subset (34/88, 39%) of identified posts remarked on broader contextual factors beyond the individual- or community-level, which may affect food insecurity (Table 3). Most of these posts (32/34, 94%) were focused on COVID-19 and related hardships or changes that put households at greater risk of food insecurity. The remaining posts provided information or opinions about federal-level decisions or programs such as the 2020 Census and changes to SNAP, as examples.

#### Supplementary Nontext Content

The text in included posts was often supplemented by additional methods of communication, such as images (72/88, 82%), emoticons (20/88, 23%), and/or videos (2/88, 2%). This supplementary content can serve to draw social media users’ attention and/or efficiently provide additional information (Table 3). For example, in the case of food assistance, images included photos of foods being distributed or digital flyers with logistical information about the event or program. Images and videos were also used to share pictures of people in the community who were supporting food security initiatives.

### Nature of Comments

There were similarities and differences in the themes of the comments (Table 4) when compared to the larger data set of posts. One key similarity was in the salient gratitude and support reflected by users’ comments, mirroring the optimism and community support indicated in the posts. On the one post that included critical jests about food assistance, comments echoed this sentiment. Similar jests were not present in comments on any other included posts. While there were a few posts relevant to literature-based codes (educational and promotion of event or business), there were no comments with relevant content. Another point of divergence was in self-disclosures of food insecurity experiences. A single disclosure of food insecurity within a comment was explicit about the individual’s family having gone hungry in the past.

**Table 4:** Comment codes, definitions, and example comments from 2019 and 2020.

Comment code	Definition	Example comment
Educational	Refers to educational resources to learn more about the issue of food security and/or opportunities for individuals or households to acquire education about food preparation and/or nutrition	Not applicable; no relevant content identified.
Paid event or business promotion	Provides information about businesses or events that require payment to support	Not applicable; no relevant content identified.
Self-disclosures	Mentions personal experiences of food insecurity or related food hardships experienced by user or user’s family members	*My mom said we would go hungry first, sometimes we did. Glad there were farmers in family*
Gratitude	Mentions appreciation for the actions and/or information provided in the post	*Thank you for the boxes today. I feel very blessed!*
Support	Mentions prior, current, desired, or intended financial support for the cause or organization mentioned in the post	*On our way to support local businesses that are helping the community....also, we hungry*
Interest in resources	Mentions the user’s interest in receiving the resources mentioned in the post	*This is amazing sister... I’m Gonna share*
Critical jests about food assistance	Presents information or cues specific to food assistance framed as a critique or joke	*I always get jealous when I see someone bust one [EBT* ^a^ *card] out [2 laughing with tears emoticons] I say ummmmm excuse me but how can I apply for one lol*

^a^EBT: Electronic Benefits Transfer.

### Audience Engagement

Among the analyzed posts, 74% (65/88) had some form of engagement via comments, reactions (Like, Love, Care, Haha, Wow, Sad, and Angry), or shares. Posts coded as reflecting community gratitude or support had descriptively more engagement (mean 19.9, 95% CI 11.2-28.5) in comparison to those that were not (mean 6.1, 95% CI 1.7-10.4). Engagement did not appreciably differ when all other broad code categories were compared. However, posts coded as incorporating culture into their text also had higher amounts of engagement (mean 26.8, 95% CI 12.7-40.9) than those that did not (mean 5.3, 95% CI 3.0-7.7).

## Discussion

Understanding and addressing food insecurity is a critical step in reducing the risk of cardiometabolic disease, including hypertension, heart disease, and diabetes, among NHPI cultural groups. The objective of this study was to describe the quantity, nature, and audience engagement of messages related to food insecurity posted online in community groups and organizations that serve NHPI audiences before and during the COVID-19 pandemic. Overall, there was a greater number of food insecurity–related Facebook posts during the pandemic compared to before the pandemic. The majority of identified posts focused on food assistance, including sharing information about resources, time-sensitive updates about services, and opportunities to support these initiatives. Cultural values of children’s food security and maintaining NHPI culture were reflected by the quantity of posts and related engagement. Broadly, rhetoric reflected sentiments of gratitude and use of humor to discuss the sensitive, and potentially stigmatizing, topic of food insecurity. This study offers a sample of the discussion on Facebook and provides a unique comparison of food insecurity discussions before and during the COVID-19 pandemic. Future work should capitalize on social media as a potential avenue to reach the unique NHPI cultural group experiencing inequitably high rates of food insecurity and risk of cardiometabolic diseases.

Keeping in mind the heterogeneity of NHPI groups with differing histories, cultural practices, and language, most of these groups faced drastic, detrimental changes after Western contact that impacted physical and mental health, which contributed to an increased risk of cardiometabolic disease and food insecurity [17,19,31,32]. The impacts of colonization continue to affect NHPI communities, with many NHPI people leaving their homelands. For example, of the 1.4 million NHPI people living in the United States, only 355,000 live in Hawai’i [33]. Given the high number of NHPI adults in the United States living away from their ancestral islands, and the elevated rates of food insecurity NHPI communities experience [17], understanding how NHPI people living in the continental United States utilize social networks to address food insecurity and promote health may help to reduce the disparities NHPI communities face. Our findings suggest that social media may be an important source of communication and connection for NHPIs, offering opportunities to share resources and bring the community together with food. For example, kalo (taro) represents the idea of a family with the main stalk representing the parent and the offshoots as the children for Native Hawaiians. There are strong beliefs in connection between food and the *‘āina* (land that feeds). Additionally, *‘ohana* (family) and a *kuleana* (responsibility) to the larger community are important values within Hawaiian culture [34]. Our findings indicating the most common posts identified were those that shared information about food assistance and engagement among other users was higher for culturally relevant posts demonstrate how Hawaiian values and culture among NHPI communities living outside of their homelands are reinforced. With geographic separation, social media may offer NHPI communities’ connections to one another to share resources, NHPI culture, and values, and thus present an opportune setting to reduce food insecurity and promote health behaviors.

Communication online related to food security and health is likely influenced by factors established in the broader communication literature. This study demonstrates how cultural values of gratitude and support were reflected in posts online. Posts that reflected these values or incorporated cultural words and references into their text had greater levels of online engagement via comments, shares, and other digital reactions. These results align with broader research in the communications field, which suggests that the way messages are presented, in their framing [35] and inclusion of emotional [36] or cultural [37] elements, can impact the success of communication strategies. Message framing has been employed for smoking cessation [38], COVID-19 vaccination [39], and dietary behavior [40] interventions, and lessons learned from these approaches should be integrated into communication interventions deployed via social media.

With the substantial increases in the use of social media, food insecurity, and the digital food environment since the onset of the COVID-19 pandemic, we explored changes in the nature and frequency of messages during and before the pandemic. Similar to research identifying changes in social media use since the onset of the COVID-19 pandemic [41,42], we identified a 10-fold increase in messages related to food insecurity in March through June of 2020 compared to the same months in 2019. The increase in posts may be related to the significant increase in food insecurity in 2020 compared to 2019 [1]. Considering the high rates of food insecurity among NHPI communities prior to the COVID-19 pandemic, these communities have also been disproportionately impacted by the pandemic; thus, recovery efforts to address the needs of NHPI communities is critical [43]. While we did not assess changes in food insecurity or behavior in this study, social media represents an important aspect of the digital food environment, and could be used to help reduce food insecurity and promote positive health behaviors such as disease management, physical activity, and cooking [12,41,44]. Future research is necessary to better understand how social media groups and pages can assist individuals, reduce food insecurity, and promote chronic disease prevention and management.

Individuals experiencing food insecurity may feel shame about their household’s circumstances [45]. Reliance on food assistance programs can be a stigmatizing experience [46] and some individuals experiencing food insecurity hesitate to use food services to avoid these feelings [47]. Thus, individuals in food-insecure households may hesitate to discuss their experiences publicly. In this study, the only public disclosure of food insecurity experiences was identified in a comment on a post, which may be seen by fewer people than the original post. This minimal personal disclosure is likely, in part, a product of the public nature of the pages and groups that were searched. The amount of self-disclosure online is related to feelings of anonymity [48] and the relationship between the people communicating [49], both of which are not often known in public online spaces. Facebook also hosts private groups, which may be perceived as a more secure online space for individuals to discuss their experiences. However, viewing these data for research would require the consent of the individuals in the groups.

Given the large presence of NHPI members in Pacific Northwest–based Facebook groups and pages, social media may provide useful platforms to provide information and social support related to food insecurity and health conditions in future interventions. Prior research describes how Facebook has been used for diabetes support groups, and particularly in the nutrition management of diabetes [12]. In fact, researchers identified that one of the most frequent topics posted on diabetes-related Facebook groups was related to food, such as preparing meals and nutrition information [12]. Therefore, the provision of nutrition information on social media may be an effective way to reach NHPI people in future initiatives. Although nutrition education was not present in this study, this may be due to the search terms used. Facebook groups and pages could partner with SNAP-Ed implementing staff to provide culturally responsive nutrition education. SNAP-Ed aims to reach and educate low-income households to shop for and prepare healthy foods [50]. Partnerships between SNAP-Ed and existing Facebook groups and pages may be particularly beneficial as SNAP-Ed uses existing frameworks and strategies related to food insecurity, while Facebook serves as a social conduit. The utility of culturally responsive interventions is evidenced by the success of programs that include cultural values and practices to reduce cardiometabolic conditions, such as the Ola Hou i ka Hula program, which saw significant improvement in hypertension management through cultural dance [20]. Culturally responsive toolkits could be efficiently scaled up to reach NHPI communities across the United States through other social media sites that attract individuals with different demographic characteristics in comparison to users of Facebook [51].

Many future research questions could build off this study. Research that seeks to ask similar questions with publicly available social media data may benefit from using community-engaged research approaches, including community members as leaders of research studies. A recent metareview found that community-engaged research can not only support culturally centered interventions but also improve the approach of observational studies [52]. Using community-engaged approaches in studies of social media data may have more comprehensive search terms, culturally informed analyses, and would be poised to inform intervention elements that are highly congruent within the contextual setting. Whenever possible, NHPI researchers should be leading these efforts or serving as crucial collaborators. However, in 2019, only 7.4% of NHPI adults have obtained graduate or professional degrees (vs 14.3% of white adults) [33], and investments to improve the representation of NHPI individuals in graduate programs are necessary to ensure equitable representation on teams investigating NHPI-related research questions. Separately, researchers may consider investigating similar questions within closed Facebook groups (with IRB approval), on other social media platforms, or in comparison with groups or pages targeting other racial and ethnic groups. Interventions using social media could have a substantial reach, and thus developing and deploying culturally tailored toolkits while studying their effectiveness at improving health-related behaviors and cardiometabolic risk factors among vulnerable groups could have important impact.

This study was not without limitations. First, qualitative research is inherently subjective and individual interpretations of the data vary. For example, had members of the Facebook group been a part of the study team, it would have allowed for insider knowledge to aid coding. However, researchers not belonging to any of the groups studied allowed for greater confidentiality during data extraction. Separately, as this was not an intervention, health data among users were not gathered. Instead, this study serves as a foundation for future research regarding food insecurity among NHPI populations. The study was also limited by the exclusive extraction of data from Facebook. While other social media sites such as Instagram, Twitter, TikTok, and Reddit draw varying demographics, Facebook use is the highest across gender, age, income, education level attainment, and household rurality characteristics [51]. This study was also limited to a subset of groups and pages. Thus, potentially relevant data within private or smaller public groups were missed. Nevertheless, the groups and pages selected had a large collective reach. Additionally, only 4 months of each year were assessed due to the intensive nature of data extraction. However, the time periods corresponded to periods of the most drastic change during the COVID-19 pandemic and the matched period a year prior for comparison. Lastly, results were restricted by the search terms used. To increase comprehensiveness, search terms were identified via the published literature, Medical Subject Headings terms, and consultations with an expert in the field.

Cardiometabolic diseases are prevalent and have pervasive impacts among the NHPI population, with food insecurity as a potential precipitating factor leading to multiple negative health outcomes. Social media and the digital food environment may be an important mechanism to reduce food insecurity and cardiometabolic diseases. Results suggest that Facebook pages and groups provide a setting for NHPI people to virtually gather and share food resources and reinforce NHPI cultural values, which has increased substantially since the onset of the COVID-19 pandemic. Future research will benefit from continued exploration of social media and the digital food environment as a mechanism to reduce food insecurity and reduce cardiometabolic disparities among NHPI adults. Specifically, social media should be further explored as a tool to promote health. Partnerships with nutrition-related organizations such as SNAP-Ed may help NHPI-serving organizations to disseminate culturally tailored messages about food assistance and educational materials to reduce food insecurity and improve health.

## Abbreviations

EBT: Electronic Benefits Transfer
HHAPI: Healthy Hearts Among Pacific Islanders
IRB: Institutional Review Board
NHPI: Native Hawaiian and Pacific Islander
SNAP: Supplemental Nutrition Assistance Program
SNAP-Ed: Supplemental Nutrition Assistance Program Education
WIC: Special Supplemental Nutrition Program for Women, Infants, and Children
